# Cluster Formation of Polyphilic Molecules Solvated in a DPPC Bilayer

**DOI:** 10.3390/polym9100488

**Published:** 2017-10-06

**Authors:** Xiang-Yang Guo, Christopher Peschel, Tobias Watermann, Guido Falk von Rudorff, Daniel Sebastiani

**Affiliations:** Institute of Chemistry, MLU Halle-Wittenberg, von-Danckelmann-Platz 4, 06120 Halle, Germany; xiang.guo@chemie.uni-halle.de (X.-Y.G.); christopher.peschel@chemie.uni-halle.de (C.P.); tobias.watermann@chemie.uni-halle.de (T.W.); guido@vonrudorff.de (G.F.v.R.)

**Keywords:** lipid bilayer, DPPC, bolapolyphile, diffusion coefficient, perfluorinated, molecular dynamics (MD)

## Abstract

We analyse the initial stages of cluster formation of polyphilic additive molecules which are solvated in a dipalmitoylphosphatidylcholine (DPPC) lipid bilayer. Our polyphilic molecules comprise an aromatic (trans-bilayer) core domain with (out-of-bilayer) glycerol terminations, complemented with a fluorophilic and an alkyl side chain, both of which are confined within the aliphatic segment of the bilayer. Large-scale molecular dynamics simulations (1 μs total duration) of a set of six of such polyphilic additives reveal the initial steps towards supramolecular aggregation induced by the specific philicity properties of the molecules. For our intermediate system size of six polyphiles, the transient but recurrent formation of a trimer is observed on a characteristic timescale of about 100 ns. The alkane/perfluoroalkane side chains show a very distinct conformational distribution inside the bilayer thanks to their different philicity, despite their identical anchoring in the trans-bilayer segment of the polyphile. The diffusive mobility of the polyphilic additives is about the same as that of the surrounding lipids, although it crosses both bilayer leaflets and tends to self-associate.

## 1. Introduction

Molecules which have the ability of self-assembling are of huge interest for biochemical (lipid bilayers), and nanosized materials [1]. To understand such behavior is crucial for the rational design of model and advanced systems. Many molecule types have been inserted into lipid bilayers in experiment and in simulations to understand their behavior. This ranges from early attempts by inserting alkanes into a lipid bilayer system to see where they are located inside the system [2], up to the latest investigations of fluorinated alkanes and alcohols [3]. In extend to those publications we investigated a very complex compound combining many philicities in one molecule. These so called polyphilic molecules, as the name suggests, are compounds consisting of fragments with different philicities. Special attention is paid here to polyphiles that contain a rigid rod-shaped aromatic core with opposing end groups and lateral groups with different philicities. The two end groups are typically highly polar, allowing the formation of multiple hydrogen bonds, which is only possible in the headgroup region of the bilayer and in the aqueous phase, whilst the lateral groups are alkyl, partially fluorinated or perfluorinated chains [4]. This novel class of molecules have received significant attention in recent years [5,6,7,8,9,10]. Polyphilic molecules can be used to modify the phase transitions temperature of a lipid bilayer [11] and also serve as a drug delivery agent [12,13]. Furthermore, they cause effects like compression or stretching of bilayer systems which was recently shown [11,14,15,16,17,18]. Fluorocarbon compounds are also studied for influencing the metabolism of rats [19,20] and for in vitro synthesis of lipid bilayer proteins [21].

**Figure 1:**
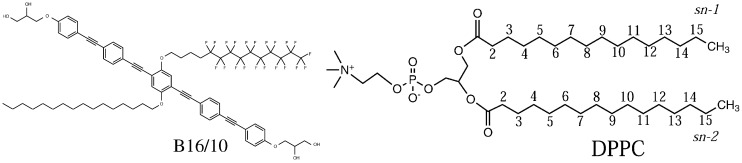
Molecular structure of the polyphilic molecule B16/10 and dipalmitoylphosphatidylcholine (DPPC) molecule studied in this report.

To fully understand why this type of molecules influences the bilayer properties, the polyphilic molecules themselves have to be studied on a molecular scale. The molecule (B16/10) being investigated in this work possesses three philicities, namely fluorophilic, hydrophilic and lipophilic parts [6,7,8,9,10,22]. The rigid aromatic phenylene–ethynylene-backbone forms its frame structure, and two hydrophilic groups are terminating this backbone chain. In the middle of the backbone, two side chains are attached, one of which is a perfluoro-*n*-alkane, the other is a regular *n*-alkane (see Figure 1). The length of the side chains can be derived from the name (16 carbon atoms for the alkyl chain and 10 carbon atoms for the perfluorinated alkyl groups). This molecular structure ensures a trans-bilayer orientation of the backbone, yielding an anchor point for the alkyl/perfluoroalkyl chains at the center of the bilayer. Mobile aliphatic side chains and polar end groups offer many possibilities of functionalization to tailor the interactions with the bilayers. As the fundamental building blocks of cellular membranes, phospholipid bilayers play a decisive role in many of their biological functions. The modification of lipid bilayer functions via interactions with biomolecules such as proteins or peptides has been widely investigated [23,24,25,26]. Due to experimental investigations of the influence of purely synthetic molecules on DPPC bilayers [14,15,27], it serves perfectly as a model system for thorough investigation of the influence of our polyphilic molecules on the bilayer properties. The concept of polyphilicity was even recently used for directly modifying the lipid bilayer itself by perfluorinating the end of the lipid tails [28].

The available experimental results show that, when bolapolyphile molecules (BP) are incorporated into gel phase lipid (DPPC) bilayers, the formation of large BP domains within the bilayer and a separation into different lamellar species can be observed [16,17]. The thermal behavior of the lipid bilayers was drastically altered upon BP incorporation and several endothermic transitions above *T*_m_ of pure DPPC bilayer occurred [11]. In the liquid phase, the BPs were homogeneously distributed in the lipid bilayer plane [11,16].

Lipid bilayer simulations have reached an exciting point, where the time and length scales of simulations are approaching experimental resolutions and can be used to interpret experiments on increasingly complex model bilayers. Within molecular dynamics (MD) of hybrid-bilayer systems, one is able to get an insight into the dynamical behavior on a molecular scale. Therefore, these simulations provide complementary information to experiments [25,29,30,31,32,33,34]. Furthermore, they can yield molecular-level insight into the structure and dynamics of these systems with a spatial resolution and time-scale that may not be feasible experimentally. Summarized, they serve as rich sources of quantitative data on molecular flexibility, lipid diffusion, ordering and atomic interactions. A detailed understanding of lipid bilayer properties is necessary to fully understand their important biophysical characteristics.

Previously, we have reported a MD study of one single B16/10 molecule inserted as a trans-bilayer agent into a dipalmitoylphosphatidylcholine (DPPC) bilayer [35]. The results showed that B16/10 is commensurate with the bilayer, and at the same time, a certain intramolecular bending and an inclination with respect to the bilayer plane is observed. While the lipophilic groups remain bilayer-centered, the fluorophilic parts tend to orient towards the phosphate headgroups, which is due to a slight size mismatch between the bilayer and the lipophilic backbone of the molecule.

In the present work, MD simulations with full atomic detail were carried out for a small trans-bilayer cluster containing six B16/10 molecules embedded into a DPPC bilayer on a large time scale. The difference of the dynamical and structural properties of the DPPC bilayer between pure bilayer system and the mixture of the lipid bilayer with B16/10 molecules is illustrated. The configurations and dynamics of incorporated B16/10 are studied. The results are compared with available experimental and literature data.

## 2. System Setup and Computational Details

**Figure 2:**
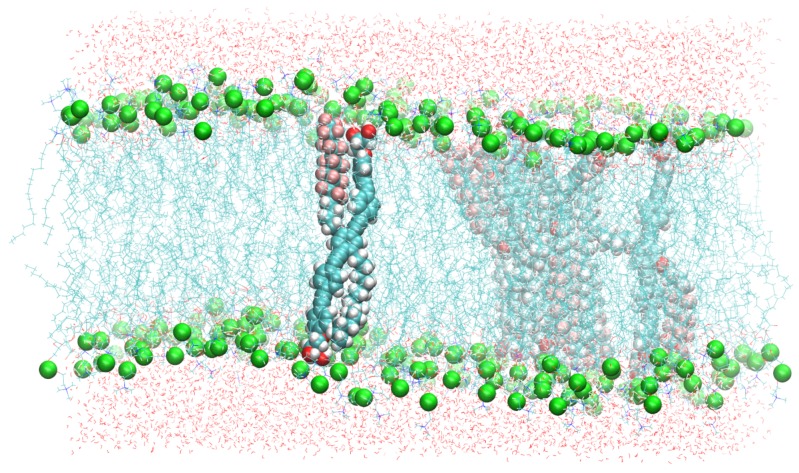
A snapshot of the simulated system containing six B16/10 molecules, 288 DPPC molecules and 8756 water molecules. Periodic boundary conditions were used in all directions. Atoms of B16/10 molecules and Phosphorus atoms of DPPC headgroups are represented by solid spheres. Phosphorus atoms are green, Fluorine atom of B16/10 side chains are pink, carbon atoms are cyan. All the lipid tails of DPPC are represented by cyan lines. One can see, that the backbone of the polyphile (cyan spheres) is bent and not straight.

A small cluster of six B16/10 molecules was embedded into a DPPC bilayer consisting 288 lipid molecules (144 per leaflet). The mixed molecular system was hydrated using 8756 TIP3 water molecules. The resulting periodic box has a dimension of $98\times 98\times 68\;{\text{Å}}^{3}$. A snapshot of the system is shown in Figure 2.

**Figure 3:**
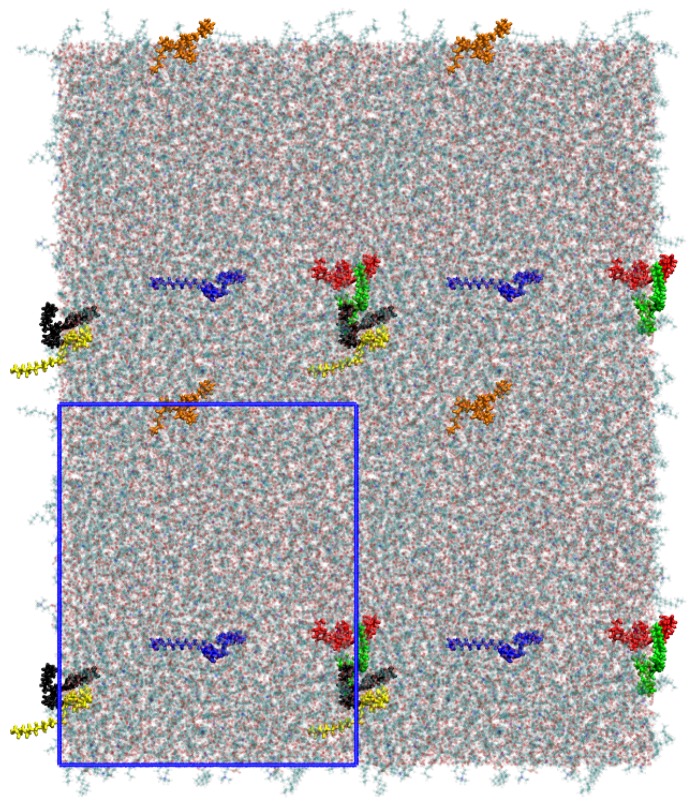
A snapshot of the simulated system as a top view after an equilibration time of 20 ns. The polyphiles are colored and a clustering can be observed for some of the molecules. The simulation cell dimensions are shown as blue rectangle.

The initial configurations of B16/10 molecules was selected based on the experimentally observed phase formation of similar structured molecules. According to the experimental results, similar structured B12 molecules spontaneously self-organize in lipid bilayers (DPPC), forming ordered snowflake like structures with 6-fold symmetry in giant unilamellar vesicles [16,17]. Furthermore, the initial structure completely vanished after an equilibration time of 20 ns. The first 20 ns have been omitted in all analyses. The structure after the equilibration is shown in Figure 3 as a top view onto the system.

All MD simulations in this work were performed by software package namd 2.9 using the CHARMM force field [32,36,37]. The detailed parameters for B16/10 are presented in a previous work of our group [38]. Three dimensional periodic boundary conditions were used. The system is kept at a constant pressure of 1 bar and a constant specified temperature (isobaric-isothermal NpT ensemble) using a modified Nose–Hoover method in which Langevin dynamics is used to control fluctuations in the barostat. The semi-isotropic pressure coupling was applied separately for the bilayer plane and bilayer normal with a coupling constant of 1 bar. Experimentally measured phase transition temperature of a DPPC bilayer is between 313 and 315 K [16,17]. It is experimentally observed that the presence of polyphile molecules increases this phase transition temperature. In order to avoid a simulation in a gel phase, we set the simulation temperature to 335 K in this work.

The system was simulated for 1 μs with a time step of 2 fs. The bond lengths were constrained using the SHAKE algorithm. The vdW cutoff follows the force field specifications for lipid bilayers. A particle mesh Ewald summation was used to calculate the electrostatic interactions. Cutoff radius for van der Waals interactions was set to 1.0 nm. Particle mesh Ewald (PME) summations were applied for long-range electrostatic interactions with a grid spacing of 0.12 nm and a cutoff radius of 1.0 nm was employed for real space summation. For the purpose of comparison, simulations of a pure DPPC bilayer was carried out under same conditions. The TIP3P water model was used to solvate the system. Data analysis of the trajectories were done by using VMD plugins [39], the python module MDAnalysis [40], the freeware program package TRAVIS [41] and our own codes.

## 3. Results and Discussion

Statistical analysis was carried out to characterize the dynamical and structural properties of B16/10 trans-bilayer molecules inside a DPPC bilayer. In particular, we (i) calculated the lateral diffusion coefficients of DPPC and B16/10 molecules and (ii) investigated the axial location and orientation of B16/10 molecules and internal structure of B16/10 cluster. The results are compared with a pure DPPC bilayer system, available experimental data and former results of our group.

### 3.1. Lateral Diffusion

**Figure 4:**
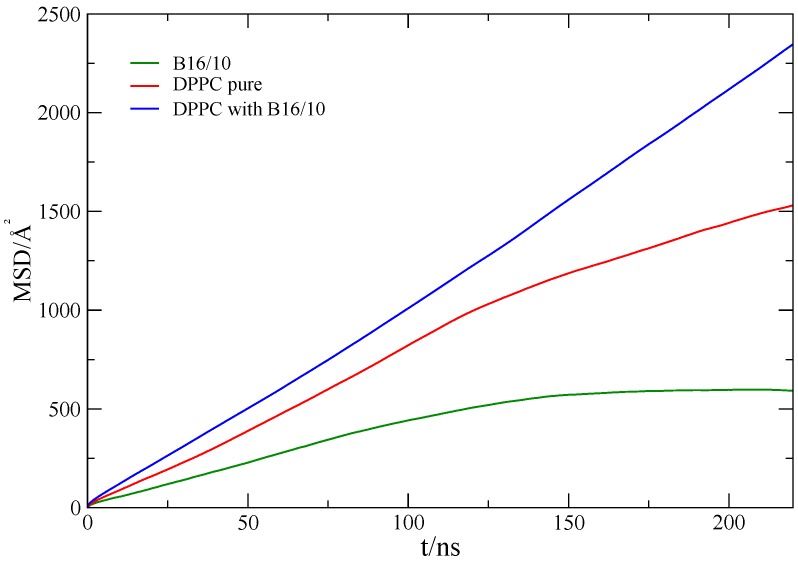
Mean square displacement of lipids in a pure DPPC bilayer (red), the lipids (blue) and B16/10 molecules (green) in the mixed system (lipids/B16/10).

Investigation of DPPC and B16/10 molecules lateral mobility in a planar lipid bilayer are carried out and the results are compared with the pure bilayer system. The averaged self-diffusion coefficients of B16/10 and DPPC molecules are calculated from the mean squared displacement (MSD) $\langle r^{2}\left(t\right)\rangle$ using the Einstein relation:

(1) $$\begin{array}{cc} MSD(t) & =\langle r^{2}\left(t\right)\rangle =\langle {\left[r\left(t\right)-r\left(0\right)\right]}^{2}\rangle \end{array}$$

(2) $$\begin{array}{cc} D & =\frac{1}{2d}\underset{t\to \infty }{lim}\frac{d}{dt}\langle {\left[r\left(t\right)-r\left(0\right)\right]}^{2}\rangle , \end{array}$$

where *d* is the number of dimension which is 2 for our calculation. The MSD is calculated with respect to the center of mass (COM) of all lipid molecules to avoid artifacts from water layer movement. The averaged MSD for DPPC and B16/10 molecules are shown in Figure 4.

The calculated self-diffusion coefficient of pure DPPC at 335K as presented in Table 1 is in very good agreement with the experimentally determined value of 14.2 ± 1.2 $\times 10^{-12}\;m^{2}/s$ [42]. The diffusion coefficient of B16/10 molecules is slightly lower than the diffusion coefficient of the lipids in a pure bilayer. For the mixed system, we find high diffusivity values for the lipid molecules, which is unexpected.

### 3.2. B16/10 Radial Distribution Functions

**Figure 5:**
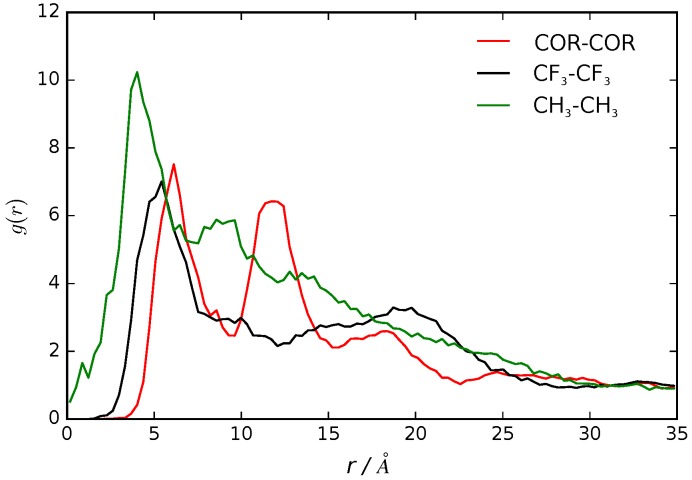
Radial distribution functions of the center of the central phenylene ring (COR), CF_3_ and CH_3_ terminal groups of B16/10 molecules within the DPPC bilayer.

We calculated the pair correlation function, g(r), as the probability of finding a pair of B16/10 molecules at distance r apart, relative to the probability expected for a completely random distribution at the same density [43]. The g(r) of the center of mass for each central phenylene ring of a B16/10 molecule (COR-COR) and of its terminal groups (CH_3_ and CF_3_) is shown in Figure 5.

**Figure 6:**
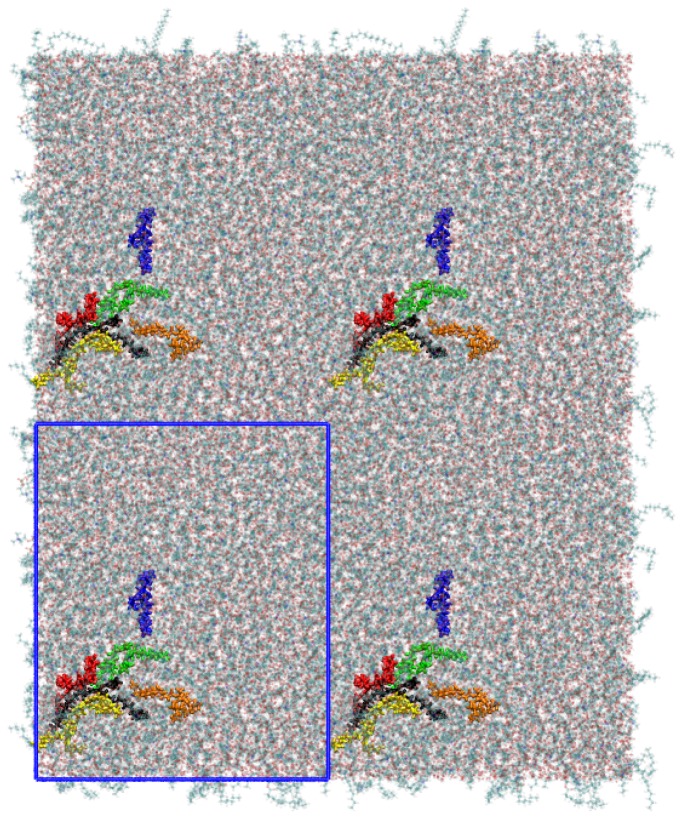
Snapshot of the top view onto the system showing the clustering of the B16/10 molecules. Namely the red, yellow and black colored molecules form a cluster, whereas the green and orange molecules are surrounding the cluster. Compared to Figure 3 (same coloring of the molecules), one can see that the cluster exchanges the molecules within the simulation. The simulation cell dimensions are shown as blue rectangle.

We observe a clustering effect documented by the two peak nature in the radial distribution function. The first peak at 7 Å distance shows that two B16/10 molecules are directly adjacent to each other, as there is no space for interlaced molecules. Nevertheless the lipid molecules force the B16/10 molecules to tilt a little away from a coplanar orientation and causing this distance of 7 Å. Due to the tilt and high distance, pi–pi stacking does not frequently occur. Nevertheless, the radial distribution function has also non-zero values below 7 Å, which at least can be interpreted as possible pi–pi stacking. As there are only six B16/10 molecules in the system, effects of a big ensemble like in experiment [11,17] cannot be captured and therefore a strong pi–pi stacking behavior cannot be excluded even though it is unfavorable in our simulation. The second peak at 12 Å shows that a third B16/10 molecule is forming a little cluster with the other two. As the second peak is not double of the first one it can be seen that they are not completely aligned but forming a triangle. The clustering can be seen in Figure 6. Comparing Figure 3 and Figure 6, one can see that the molecules inside the cluster are exchanging within the simulation.

Having this in mind it is even more surprising that the diffusion coefficient discussed in the previous section of $11\times 10^{-12}\frac{m^{2}}{s}$ for B16/10 (see Table 1) is rather high. This clustering behavior of x-shaped molecules like the non-perfluorinated B12 molecule was also observed experimentally [11,17]. Therefore it can be assumed that by perfluorinating one side chain, the clustering behavior is maintained. For the terminal groups CH_3_ and CF_3_ there is only one well defined peak at around 3 Å for CH_3_ and at 5 Å for CF_3_. Beyond this there is a smooth decay to unity apart from residual structure but with no recognizable pattern. All in all this shows that there is a correlation of the backbones of the B16/10 molecules forming a little cluster whereas there is no correlation of the side chains aside from pairwise orientation.

### 3.3. B16/10 Backbone Angles Distribution

**Figure 7:**
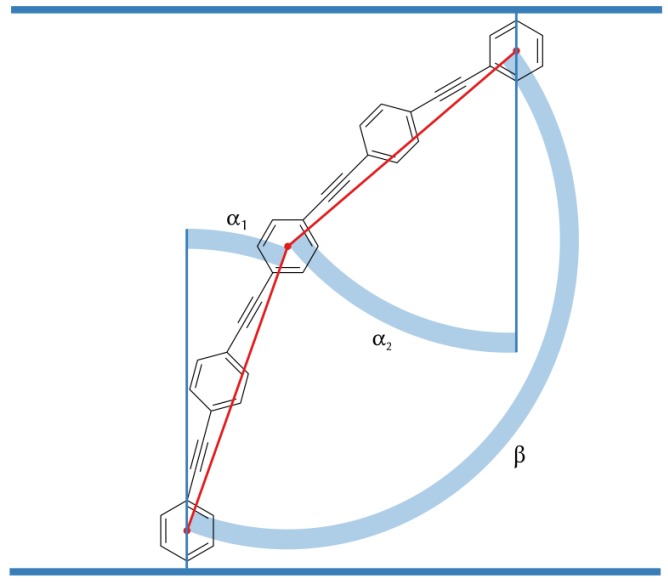
Angels between the bilayer normal and B16/10 backbone ($\alpha_{1}$ and $\alpha_{2}$) and backbone bending angle β.

**Figure 8:**
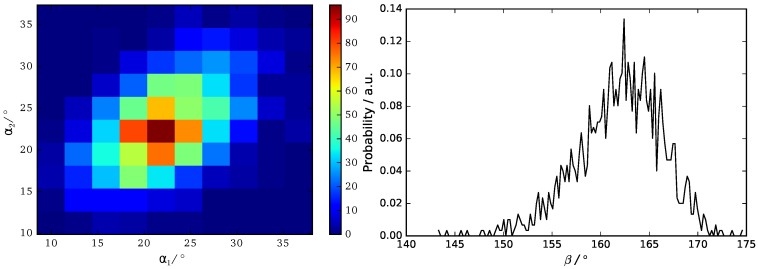
Probability distribution of backbone angles $\alpha_{1}$, $\alpha_{2}$ and β.

We use three angels $\alpha_{1}$, $\alpha_{2}$ and β to characterize the orientation of B16/10 backbone within the bilayer as shown in Figure 7. The distributions of the three angels are displayed in Figure 8.

The calculated angular distributions of the B16/10 backbone are in very good agreement with our former results of a single B16/10 molecule inside a DPPC bilayer [35]. The B16/10 molecules are well incorporated into the lipid bilayer, cross the whole bilayer with their well-matched hydrophobic core length and adapting into the bilayer by a slight tilt.

### 3.4. B16/10 Terminal Group Integration and Side Chain Orientations

**Figure 9:**
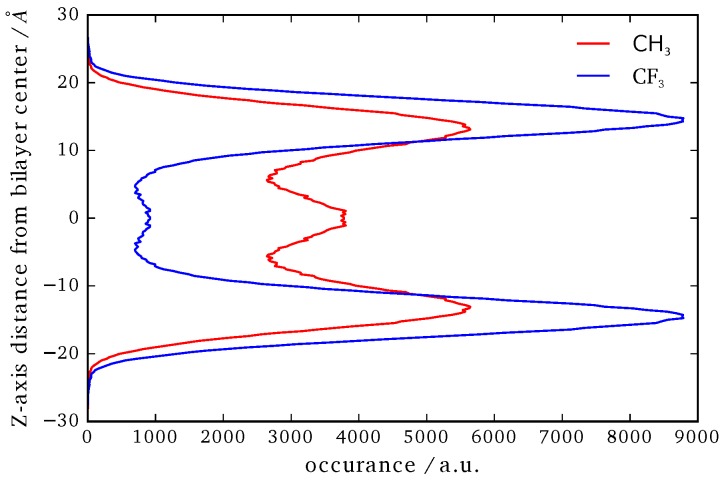
Position of the CF_3_ and CH_3_ terminal groups along the *z*-axis relative to the center of mass of the whole system.

In Figure 9 the averaged position distribution of CF_3_ and CH_3_ terminal groups of B16/10 molecules relative to the center of mass of the system is shown. For avoiding artifacts, the center of mass movement of the whole system has been subtracted. The center of the lipid bilayer is denoted by z=0 and *z* is the axis along the normal of the bilayer plane. The bilayer itself has a thickness of approximately 40 ± 2.5 Å. As one can see in Figure 9, both side chains approach the head group region of the bilayer. Nevertheless the CF side chain tends to stay more in the headgroup region than the CH chain. The combination of higher solubility inside the leaflets (which was recently shown by Brehm et al. [3]) and sterical issues force the CF_3_ terminal group to stay in the headgroup region as in direct comparison the CF side chain is nearly inflexible whereas the CH side is more flexible and thinner which makes it easier for the CH chain to find its way through the lipid bilayer. Not surprising is, that the CH_3_ terminal group has a high occurrence in the middle of the bilayer, as this the most non polar region of the bilayer which has been observed in experiment before for alkanes [2]. Of course sterical issues are more dominant because not every polyphile can arrange like this when incorporated into a cluster and therefore it also stays frequently inside the leaflets. On first sight the occurrence of the CF_3_ terminal group in the middle of the bilayer seems astonishing. It is easy explained by attempts to flip the sides of the bilayer. As it is only ninth of the occurrence of the highest peak this rarely happens and is never a stationary state. By closer inspection we found that the flipping of the CF side chain happens on a nanosecond time scale.

**Figure 10:**
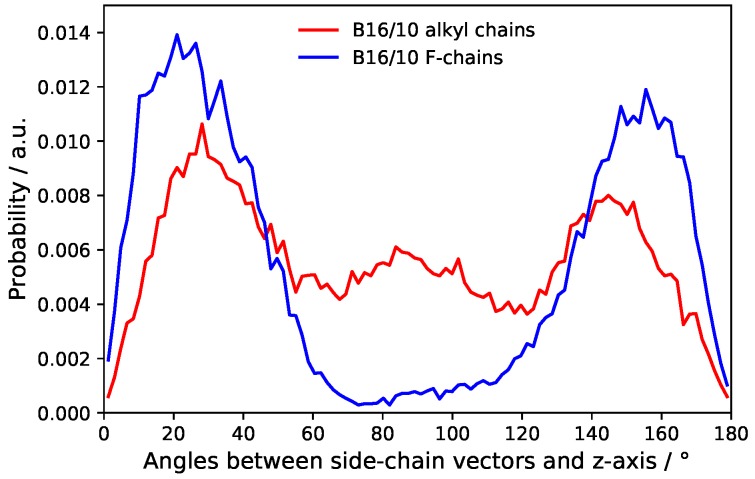
Probability distribution of side chains angles.

The distribution of the side chain angles can be used to confirm these statements as seen in Figure 10. The angles are defined as the angle of the vector from the terminal groups to the center of the phenylene ring in the middle of the backbone and the bilayer normal. Therefore 90° denotes directly aligned between the leaflets in the bilayer plane. The prominent angles around 30° and 150° for the CF_3_ terminal group confirms that it mostly stays in head group region of the bilayer. The very slight occurrence around 90° also shows the behavior to flip rather than staying between the leaflets. More or less Figure 9 and Figure 10 resemble each other in appearance and therefore also confirm the statements for CH terminal head group mentioned above.

## 4. Conclusions

In this paper, we report a study of six polyphilic molecules embedded in a lipid bilayer/water system. Our results give insights not only into the the conformational preference of the B16/10 molecules inside the DPPC bilayer, but also on the dynamics of the B16/10 molecules in the DPPC bilayer environment. Within 1 ms of simulation the stability of the bilayer maintained upon insertion of the additive B16/10 molecules. The diffusion of the lipid molecules is increased compared to the pure lipid bilayer and B16/10 molecules move only slightly slower than the lipids in the pure bilayer. For the intermolecular structure of the B16/10 molecules a clustering effect can be observed in the RDFs. The difference in the side chain orientation and configuration is also in good agreement with the simulation results we obtained before [35]. In general, the conclusions drawn from simulations are consistent with experimentally observed effects [16,17,42]. We anticipate that these findings will be important for understanding the role of polyphilic molecules in modulating and modifying bilayer properties.

## Figures and Tables

**Table 1:** Lateral self-diffusion coefficients (*D*) at 335 K obtained from MD simulation (MD) and experimental data from literature (exp) [42]. Uncertainties are given as three times the standard deviation.

*D*/($10^{-12}\;m^{2}/s$)	Lipids (Pure DPPC)	Lipids (mix)	B16/10 (mix)
MD	13.9 ± 0.3	24 ± 0.6	10.7 ± 0.3
exp	14.2 ± 1.2		
